# Structural Brain Alterations Associated With Alcoholism

**Published:** 1995

**Authors:** Margaret J. Rosenbloom, Adolf Pfefferbaum, Edith V. Sullivan

**Affiliations:** Margaret J. Rosenbloom, M.A., is a health science specialist in the Psychiatry Service, Veterans Affairs Palo Alto Health Care System, Palo Alto, California, and in the Department of Psychiatry and Behavioral Sciences, Stanford University, Stanford, California. Adolf Pfefferbaum, M.D., is chief of psychiatric research, Veterans Affairs Palo Alto Health Care System, and a professor in the Department of Psychiatry and Behavioral Sciences, Stanford University, Stanford, California. Edith V. Sullivan, Ph.D., is an associate professor in the Department of Psychiatry and Behavioral Sciences, Stanford University, Stanford, California, and a health science specialist in the Psychiatry Service, Veterans Affairs Palo Alto Health Care System, Palo Alto, California

**Keywords:** brain, AOD dependence, heavy AOD use, chronic AODE (alcohol and other drug effects), neuroimaging, magnetic resonance imaging, computed x-ray tomography, risk factors, AOD abstinence

## Abstract

Structural changes in the brains of chronic heavy drinkers that were first observed in pathological studies have been supported and expanded upon using computed tomography (CT) and magnetic resonance imaging (MRI) techniques. In general, the volume of brain tissue appears decreased in chronic drinkers, and this finding may be affected by a person’s age, gender, and other factors. MRI studies also demonstrate some increase in brain tissue volume after a chronic drinker has been abstinent for a period of months. Whether this tissue increase can be linked with recovery of brain functioning remains unanswered.

The neuroimaging techniques of computed tomography (CT) and magnetic resonance imaging (MRI) provide noninvasive ways to examine the structure of the living brain. Using these techniques, investigators have shown that many people with histories of heavy alcohol consumption^1^ have brain structures that differ markedly from people without such histories. These structural changes may affect the higher brain functions of heavy drinkers, such as short-term memory and problem-solving. Scientists do not yet know, however, the mechanisms by which an alcohol-related structural change may alter brain function. Researchers in the imaging field are taking an initial step toward answering this question by defining the patterns of brain changes that most chronic alcohol abusers experience. Such patterns may help link structural damage to specific functional deficits.

This article briefly describes brain-structure abnormalities found in alcoholics through the use of CT and MRI and provides partial answers to the following related questions that are important to consider when treating alcoholism: Are some alcoholics at greater risk than others of developing structural brain damage? What are the chances that the brain will recover its normal structure once abstinence is attained? And how do structural brain changes translate into functional problems?

## Structural Brain Changes in Alcoholism

### Early Pathological Studies

Pathological studies were the first to demonstrate an association between heavy alcohol consumption and structural changes in the brain. In contrast to CT and MRI studies, pathological studies examine the brain after death (i.e., post mortem). Early studies of patients with histories of heavy alcohol consumption showed a generalized shrinkage of the brain, sometimes more pronounced in the frontal lobes^2^ (Courville 1955). Post mortem studies measuring the amount of different tissue types on individual slices of the brain showed that of the two primary types of brain tissue, the fiber-containing white matter, but not the gray matter (which is made up mainly of nerve cell bodies), is reduced in volume in alcoholics. Thinning of the corpus callosum, the bundle of white-matter fibers connecting the left and right cerebral hemispheres, is an example of white-matter reduction in alcoholics. Although post mortem studies have not shown a general reduction in gray-matter volume, some microscopic post mortem research has demonstrated reductions in the size of individual gray-matter cells, loss of cells, and less branching of fibers off the cells in some brain areas (Harper and Kril 1990).

Subjects used in pathological studies who had had specific neurological illnesses frequently associated with excessive alcohol use, such as Korsakoff’s syndrome (a condition involving the inability to remember new information), also showed reduced size or other abnormalities in specific brain structures beneath the cortex (i.e., subcortical structures) that are involved in memory and motor activities. These structures include the mammillary bodies, the thalamus, and the cerebellar vermis, which lies between the two cerebellar hemispheres (Charness 1993). Other pathological studies also have demonstrated a loss of cell bodies as well as a reduction in size of the hippocampus, a part of the brain that plays an important role in memory (Bengochea and Gonzalo 1990).

### Structural Changes Seen Through Imaging

**Figure 1:**
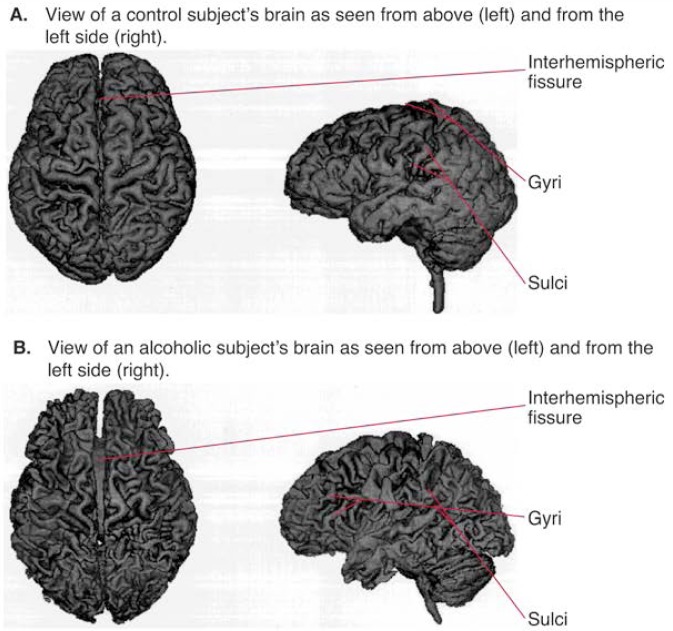
Three-dimensional renderings of the brains of two men at age 63. To produce these images, scientists digitally peeled away the scalp, skull, and cerebrospinal fluid to reveal the grooves (i.e., sulci) and ridges (i.e., gyri) marking each brain’s external surface. These images of living brains are comparable to post mortem photos of brains for pathological study. Brain A is from a healthy social drinker who has an estimated lifetime consumption of 54 kilograms (kg) of pure alcohol. Brain B is from a person who reported a history of heavy drinking over the past 32 years and who has an estimated lifetime consumption of 1,882 kg of pure alcohol. The shriveled appearance (i.e., wider sulci and narrower gyri) of brain B sharply contrasts with brain A’s relatively plump appearance (i.e., well-filled gyri and narrower sulci) and reflects the tissue shrinkage associated with heavy drinking. Also, tissue shrinkage has widened the interhemispheric fissure in brain B, exposing the bundle of fibers connecting the two hemispheres (i.e., the corpus callosum). In contrast, the corpus callosum can barely be seen in brain A.

Brain structures measured post mortem do not necessarily reflect how those structures appeared when the person was still alive. Investigators have therefore used in vivo imaging techniques—first CT and more recently MRI—to examine the brain in living subjects (details about these techniques are provided in the article by Doria, pp. 261–265). CT technology yields images showing vertical slices of the brain as if they were cut from the bottom to the top (i.e., in an axial plane) and in which the cerebrospinal fluid (CSF) appears dark and the tissue appears light. In contrast, magnetic resonance images of the brain can be obtained from different angles, allowing axial views (like CT) as well as views from side to side and back to front. Magnetic resonance images discriminate not only between CSF and tissue but also between white and gray matter. Another advantage of MRI is that images can be acquired in three dimensions and then sliced to display specific internal brain structures or processed to display the brain’s exterior surface (figure 1). Images of both internal slices and the external surface can be created from any angle.

**Figure 2:**
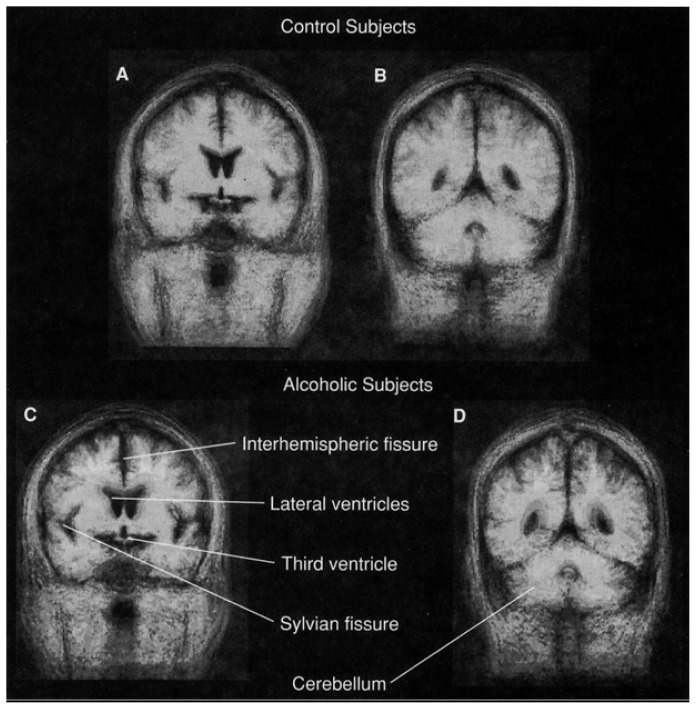
Images of “average” brains, combining the individual magnetic resonance images of 20 male, nonalcoholic control subjects (A and B) and 16 male, alcoholic subjects (C and D) age 45 or older. The dark areas represent regions where cerebrospinal fluid (CSF) exists in all group members. The gray areas represent regions where CSF exists in some, but not all, group members. Images A and C are of a slice taken from the front part of the brain that cuts through the Sylvian fissure, the body of the lateral ventricles, and the third ventricle. The fissure separating the brain’s two hemispheres also can be seen clearly. Images B and D show a slice from the back part of the brain cutting through the hindmost sections (i.e., horns) of the lateral ventricles and through the cerebellum. The images clearly show the overall enlargement of CSF-filled spaces in the alcoholic group compared with the control group.

New advances in image processing have enabled magnetic resonance brain images of each member of a study group to be adjusted to the same scale and angle and then combined to form a composite, or “average,” brain for that group. This technique can be used, for example, to visually compare a group of healthy men with a group of alcoholic men and to identify brain areas that differ between the two groups (figure 2). Areas appearing to be different may then be measured separately on each subject’s brain image and statistically compared to determine whether the variation is significant.

### Accounting for Individual Differences

**Figure 3:**
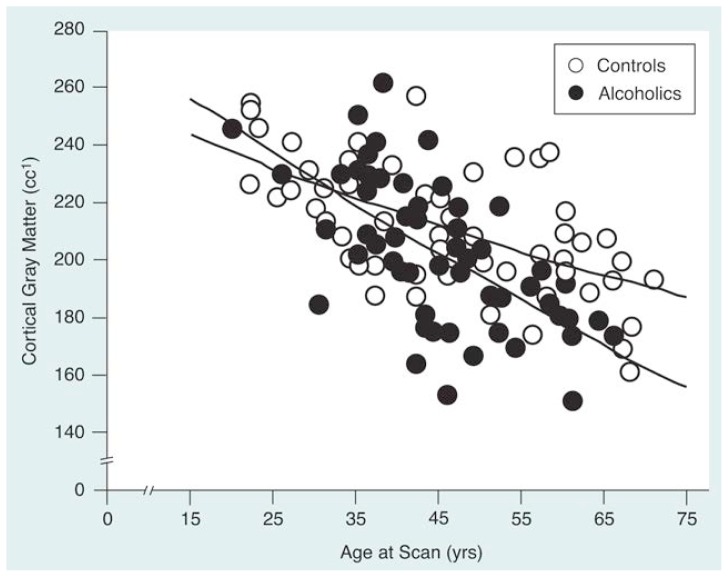
A comparison of the amount of gray matter in the brain cortex among 58 healthy nonalcoholic men (open circles) and 57 alcoholic men (closed circles) plotted by age. For both groups, the amount of gray matter decreases with age; the rate of decline, however, is steeper in the alcoholics than in the nonalcoholics (i.e., the control subjects). Although considerable overlap exists in the range of gray-matter volumes between the two populations, the alcoholic men as a group have less gray matter than the control men, a difference that becomes increasingly apparent in older subjects. ^1^cc = cubic centimeters.

Both CT and MRI studies have shown that, on average, patients who meet the criteria for alcohol dependence show larger volumes of CSF and smaller volumes of brain tissue when measued against control groups of healthy people who abstain or are very light social drinkers. Individual differences do exist, however, and some alcoholics may be indistinguishable from many control subjects. In fact, some alcoholic subjects may even have larger brain-tissue volumes than do some control subjects (figure 3). Only by testing many subjects and making statistical comparisons that take individual variability into account can the particular differences attributable to the disease be identified.

### Findings Using CT and MRI

CT studies have shown that CSF-filled spaces in the middle and lower parts of the brain (i.e., the lateral and third ventricles) are often enlarged in chronic alcoholics (Lishman 1990; Wilkinson 1985), as are the fissures (i.e., sulci) located on the surface of both the brain (i.e., the cerebrum) and the cerebellum, or “little brain,” which lies at the back of and below the cerebrum (Haubek and Lee 1979; Hillbom et al. 1986). The enlargement of CSF-filled spaces has been viewed as evidence of shrinkage or atrophy of the adjacent brain tissue. MRI technology has confirmed findings seen with CT. In addition, MRI has shown that cortical volumes of both gray (Jernigan et al. 1991; Pfefferbaum et al. 1992) and white (Pfefferbaum et al. 1992) matter are reduced in alcoholics and has confirmed structural changes previously only seen in post mortem studies. These alterations include reduced volumes of sections of the brain involved with muscle control, balance, and coordination, such as the cerebellar hemispheres and the cerebellar vermis (Davila et al. 1994). Researchers also have detected reductions in subcortical structures involved in memory, such as the front (i.e., anterior) portion of the hippocampus (Sullivan et al. 1995*a*) and the mammillary bodies (Blansjaar et al. 1992; Davila et al. 1994). In previous pathological studies, damage to the mammillary bodies has been found typically in alcoholic patients with severe memory loss associated with Korsakoff’s syndrome. In vivo MRI studies, however, have demonstrated that these structures are compromised even in alcoholic patients who do not exhibit severe memory problems. Finally, MRI recently has confirmed pathological studies showing reduced corpus callosum size in alcoholics (Hommer et al. 1995; Pfefferbaum et al. in press).

## Susceptibility to Structural Brain Changes

Although researchers can discern patterns of alcohol-related structural brain changes, certain personal characteristics, such as age and gender, appear to affect an alcoholic person’s risk of developing brain abnormalities.

### Age

As healthy people age, they undergo substantial changes in a number of brain structures that also are affected by alcoholism. For example, in normal aging, the percentage of the brain that is made up of cortical gray matter declines from approximately 46 percent in the early twenties to approximately 35 percent by the seventies (Pfefferbaum et al. 1994). One challenge in interpreting MRI and other in vivo neuroimaging data from alcoholics is to distinguish changes that occur with normal aging from changes that may be attributed to alcoholism. To measure the extent and types of normal age-related changes, researchers have measured the brains of large groups of healthy men and women, ranging in age from their early twenties to early seventies, who are abstainers or light social drinkers. The scientists have developed age “norms” for different brain measures in these healthy subjects.^3^ Consequently, the scientists can compare individual alcoholic patients with the age norms to assess the effects of alcoholism independent of the effects of the normal aging process.

Most brain structures measured by Pfefferbaum and colleagues (in press, 1992, 1988) and Sullivan and colleagues (1995a) (i.e., fluid in the sulci and ventricles; cortical gray and white matter; and the hippocampus, corpus callosum, cerebellum, and cerebellar vermis) show that older alcoholic men have more pronounced abnormalities for their age than do younger alcoholic men (figure 3). This finding does not appear to result only from the older alcoholics’ greater number of years of heavy drinking. One study sample included older alcoholics who started drinking heavily relatively late in life. Their brains were more extensively altered compared with nondrinkers of the same age than were the brains of younger alcoholics even though the lifetime alcohol consumption of the older alcoholics resembled that of the younger alcoholics. Thus, the aging brain appears especially vulnerable to alcohol’s effects. Studies from other laboratories support this hypothesis, having either failed to find a difference in structural brain measures between samples of young alcoholic and nonalcoholic men (Wang et al. 1993) or having found that deficits were more pronounced in older than in younger alcoholics (Hayakawa et al. 1992).

### Gender

Gender also may affect a person’s susceptibility to structural brain changes resulting from heavy drinking. Few investigators have designed studies specifically to investigate whether the types of structural brain changes found in alcoholic men occur in alcoholic women. Two studies of alcoholic women that were conducted using CT found that after taking into account normal gender differences in head size (women typically have smaller heads than men), alcoholic women had equivalent levels of general brain shrinkage compared with age-matched alcoholic men (Jacobson 1986; Mann et al. 1992). In both studies, however, the men had been drinking heavily for more years than the women had, suggesting that women develop structural changes in response to less alcohol consumption. Another study, showing that women had levels of cognitive dysfunction equivalent to those of men after fewer years of alcohol dependence, supported this hypothesis (Glenn 1993). Together these findings have led to the hypothesis that women are more vulnerable than men to alcohol-related structural brain damage. Additional studies using gender-specific norms for age and head size need to be conducted to fully examine gender differences in the nature and extent of structural brain changes associated with women’s typically lower levels of alcohol consumption.

### Other Factors Affecting Brain Structure in Alcohol-Dependent Patients

Women and older men may be at increased risk of experiencing brain alterations from the effects of alcoholism. In addition, patients who suffer severe withdrawal after stopping heavy drinking appear to show more structural brain deficits than those who stop drinking with relative impunity. Recent studies have reported that patients who suffer withdrawal seizures (Sullivan et al. in press) or “the shakes” (i.e., delirium tremens) (Daryanani et al. 1994) show greater brain changes than those who do not.

Some medical conditions associated with chronic alcohol dependence, such as extreme malnutrition or head injuries resulting from trauma, can themselves alter the brain’s structure. The extent to which these conditions leave the brain vulnerable to alcohol’s toxic effects has not yet been well studied. Even among relatively well-nourished chronic alcoholics, however, measures of nutritional status, such as those reflected by anemia, have been found to correspond to the amount of enlargement in brain ventricles seen in alcoholic patients (Pfefferbaum et al. 1992, 1988).

## Mechanisms of Structural Brain Changes

MRI, generally consistent with CT and post mortem studies, has revealed that certain brain structures are altered as a result of heavy drinking, and personal characteristics appear to affect an alcoholic’s susceptibility to these changes. The action by which alcohol causes structural changes, however, is not known. One possibility is that the alterations result from the direct toxic effects of alcohol on the brain or of the compounds that result when alcohol is metabolized. Another theory is that structural changes result indirectly from conditions associated with alcoholism, including malnutrition, head injury from trauma, liver disease, or severe withdrawal syndromes. An additional possibility is that the structural brain abnormalities seen in chronic alcoholics are preexisting conditions that predispose people to become alcohol dependent. This hypothesis is less likely, however, because these brain abnormalities are not seen in young alcoholics who are in the early stages of their disease; they also are not seen in nonalcoholic relatives or in the identical twins of alcoholics (Gurling et al. 1984). Moreover, the structural changes may show some recovery with abstinence.

## Functional Significance of Structural Brain Changes

Alcoholics commonly suffer mild, yet significant, cognitive deficits involving planning, organizing, problem-solving, and abstraction (i.e., executive functions); short-term memory; verbal fluency; and the capacity to deal with objects in two- or three-dimensional space (i.e., visuospatial abilities) (for a review, see Parsons 1987). Such cognitive impairments may be attributable to the structural brain changes observed using MRI. Despite much effort, however, investigators have not yet demonstrated meaningful, predictable associations between cognitive deficits and brain-structure loss. Although studies have been unable to demonstrate how structural brain changes relate to complex cognitive operations, the association between structural changes and more simple sensory and motor processes has been demonstrated. For example, studies have shown that alcoholic patients have a decreased sense of smell (i.e., olfaction) (DiTraglia et al. 1991) and lose their ability to maintain balance (Sullivan et al. 1995*b*). These deficits have been related, respectively, to the volume of the thalamus—a structure involved in the brain circuits that process odors—(Shear et al. 1992) and the anterior superior vermis—the part of the cerebellum involved in balance and coordination (Davila et al. 1994; Sullivan et al. 1996). These findings suggest that an obvious association does not exist between structural brain changes and cognitive deficits.

Scientists’ failure thus far to establish structure-function relationships for complex cognitive operations may result from limitations in the types of brain-imaging measures currently available. That is, the existing measures may not be sensitive or specific enough to characterize the underpinnings of complex cognitive behaviors. Furthermore, if subjects are examined only once, imaging techniques cannot capture the dynamic interplay over the course of a chronic illness between structural brain changes and their behavioral antecedents or consequences. Brain-function deficits may even precede measurable structural change. For example, some studies have indicated that alcoholics experience reduced metabolic activity in their brains—suggesting that less than normal “processing” occurs—even in the absence of structural change (Wang et al. 1993). Long-term studies tracking changes in brain structure and cognitive performance will help clarify the association between structural changes and deterioration in cognitive functioning.

## Time Course of Structural Brain Changes

Many important questions remain unanswered regarding how long a person can drink heavily before brain changes occur. Scientists do not know whether brain structure can fully regenerate with abstinence and whether younger people have a greater chance of experiencing structural regeneration than older people. Perhaps the most important question is whether recovery of brain structure (i.e., measured in the MRI studies below as increased volume of gray matter or white matter) is accompanied by the restoration of cognitive abilities.

### Challenges to Methodology

To answer these time-course and recovery questions would require the long-term study of a group of subjects. Such studies are difficult to conduct with humans. Researchers must consider that their subjects exhibit a wide range of confounding demographic, medical, and psychiatric characteristics; live varying lifestyles; and drink when and what they want. Over time these uncontrolled characteristics affect patients in different ways, hampering researchers’ ability to separate alcohol’s effects on the brain from the effects of these other variables.

One approach to studying long-term questions has been to infer effects over time by comparing patients of different ages and with different ages of onset, lengths of illness, or lengths of alcoholism remission. Variability can be confined by limiting a study to patients who are within a certain age range, have specified histories of alcohol use, do not have co-occurring illnesses, or are in hospital settings where access to alcohol is restricted. However, these patients’ length of drinking history, age of onset, and ongoing drinking behavior can be manipulated experimentally only in a restricted fashion. Another approach is to conduct a long-term study in which patients who have not been assigned to specific study regimens (i.e., a naturalistic study) are followed over time and retested at intervals. When comparing brain images obtained at different times from the same person, researchers must consider that apparent brain differences can result as much from differences in how patients’ heads are positioned or other factors during scanning as from real structural changes brought about by continuation or cessation of alcoholic drinking. To control for this type of error, scientists must test and retest nondrinking subjects at intervals similar to those used in tests conducted on heavy drinkers.

### Findings From Long-Term Studies

Despite methodological difficulties, results from long-term brain imaging studies have been reasonably consistent (Pfefferbaum et al. 1995; Shear et al. 1994; Zipursky et al. 1989). With abstinence, both gray- and white-matter tissue volumes increase, although the changes may occur at different rates in each. In one study (Zipursky et al. 1989), alcoholic patients were first scanned within 10 days of their last drink and again after 3 to 4 weeks of abstinence. These patients’ ventricles were statistically smaller at the time of the second MRI, indicating that some tissue loss can be reversed quite rapidly. A second study confirmed and extended this finding (Pfefferbaum et al. 1995). Patients were scanned when they entered treatment and again after completing 28 days of rehabilitation. A subgroup then was retested 2 to 12 months after discharge from the program. At that time, approximately 50 percent of the patients had remained abstinent; the other 50 percent had resumed drinking. Nonalcoholic subjects were retested at the same intervals as the alcoholic patients to provide a control group. After 4 weeks of abstinence, the size of the lateral ventricles within the brain and the sulci on the posterior surface of the brain had decreased in the alcoholic patients, and the amount of gray matter in certain regions had increased compared with the measurements taken at 10 days. After a longer interval (i.e., between 2 and 12 months), patients who remained abstinent showed even greater reduction in ventricle size and had stable white- and gray-matter volumes, whereas the alcoholics who resumed drinking showed further loss of white matter and expansion of the third ventricle. These data suggest that gray-matter changes and changes in the sulci and lateral ventricles occur early in the course of abstinence, whereas white-matter and third-ventricle volumes show improvement later during abstinence. Other scientists also have observed white-matter (Shear et al. 1994) and third-ventricle (Muuronen et al. 1989) improvement with extended abstinence.

## Summary

CT and MRI observations of the brains of chronic alcoholics indicate widespread loss of brain tissue. Older men are more vulnerable to the toxic effects of alcohol on the brain, particularly in the frontal lobes, than are younger men, and women may be more vulnerable than men, although systematic studies of women are only beginning. Withdrawal seizures, nutritional deficiencies, and other medical conditions associated with alcoholism appear to exacerbate some of these structural brain changes. Detailed imaging of the brain has revealed size and other deficits in a number of specific structures deep in the brain (e.g., the mammillary bodies, the hippocampus, and the corpus callosum). Deficits in the size of certain brain structures are found even in alcoholics without extreme symptoms, such as Korsakoff’s syndrome, to which such structural loss previously had been linked. Although researchers have not linked changes throughout the cortex and in the hippocampus to cognitive behavioral function using current testing strategies, structural deficits in other areas (i.e., the cerebellum) are related to difficulties with balance. Likewise, deficits in the thalamus are correlated with an impaired sense of smell.

Studies of alcoholic patients conducted over the course of their illnesses have indicated that after relatively brief periods of abstinence (e.g., 4 weeks), certain areas of the brain will recover. Longer periods of abstinence (e.g., more than 3 months) are associated with additional recovery in brain structures (i.e., reduction of third-ventricle volume). White matter appears to be especially vulnerable to damage incurred by the resumption of drinking after a period of abstinence. Although investigators do not know whether recovery of gray-matter volume stemming from abstinence signals restoration of cognitive function, this relationship may yet be documented.

## Footnotes

1 In this article, the term “heavy” alcohol consumption refers to more than 80 grams of pure alcohol or approximately six standard drinks per day. The term “alcoholism” as used by the authors in reference to their own work means alcohol dependence as defined by the American Psychiatric Association’s *Diagnostic and Statistical Manual of Mental Disorders, Third Edition, Revised*. Otherwise, the definitions of “alcoholism” and “alcohol abuse” are based on the terms used in the references cited.

2 For the locations and descriptions of these and other brain structures, see glossary and diagrams, pp. 293–295.

3 ”Norms” of brain values are derived from analyses of numeric values for certain brain measures related to a subject’s age, whereas average brains are simply visual compilations of images from several subjects.
